# Identification of the *czc* metal efflux operon on a new plasmid type in a *Pseudomonas aeruginosa* clinical isolate belonging to ST357 O11

**DOI:** 10.1093/jac/dkaf058

**Published:** 2025-03-05

**Authors:** Adam Valcek, Diana Isabela Costescu Strachinaru, Patrick Soentjens, Anke Stoefs, Charles Van der Henst

**Affiliations:** Microbial Resistance and Drug Discovery, VIB-VUB Center for Structural Biology, VIB, Flanders Institute for Biotechnology, Brussels, Belgium; Structural Biology Brussels, Vrije Universiteit Brussel (VUB), Brussels, Belgium; Center for Infectious Diseases, Queen Astrid Military Hospital, Brussels, Belgium; Center for Infectious Diseases, Queen Astrid Military Hospital, Brussels, Belgium; Department of Clinical Sciences, Institute of Tropical Medicine, Antwerp, Belgium; Department of Microbiology, Queen Astrid Military Hospital, Brussels, Belgium; Department of Microbiology, Universitair Ziekenhuis Brussels, Brussels, Belgium; Microbial Resistance and Drug Discovery, VIB-VUB Center for Structural Biology, VIB, Flanders Institute for Biotechnology, Brussels, Belgium; Structural Biology Brussels, Vrije Universiteit Brussel (VUB), Brussels, Belgium

*Pseudomonas aeruginosa* is an opportunistic bacterial pathogen and a major concern in burn units worldwide.^1^ As a member of the ESKAPE group, *P. aeruginosa* represents a global threat to human health. *P. aeruginosa* has various intrinsic resistance mechanisms, such as resistance-nodulation-division pumps involved in the efflux of toxic substances, including heavy metals and antibiotics.^2^

In October 2022, an explosion victim was admitted to the Burn Unit of the Queen Astrid Military Hospital in Brussels (Belgium) after 15 days in a Romanian hospital. Both *Acinetobacter baumannii* and *P. aeruginosa* were isolated from rectal and wound swabs and an endotracheal aspirate on admission and later also from central line catheter and blood cultures (only *A. baumannii*). Three *Pseudomonas* isolates from endotracheal aspirate (0056T; 12 days after admission), central line catheter (0430T; 49 days after admission) and rectal swab (0543T; 104 days after admission) were subjected to long-read whole-genome sequencing using the PromethION (P2 Solo) platform by Nanopore (GenBank BioProject PRJNA1111012). Multi-locus sequence typing (https://github.com/tseemann/mlst) identified 0056T as ST357 O11, a high risk epidemic clone^3^ and 0430T and 0543T as the same single locus variant of ST357 (*aroE*444; D182N G544A versus *aroE*4 in ST357). All three isolates carried a 151 111 bp plasmid (99.98% nucleotide identity), designated p0056T, p0430T and p0543T, carrying a *czcCBA* operon, identical to that of pNK546a, involved in Ag^2+^, Cu^2+^, Co^2+^, Cd^2+^ and Zn^2+^ efflux.^4^

A *czc* operon (*czcABC*) is often found on the chromosome or a megaplasmid in *P. aeruginosa* and is often associated with antibiotic resistance genes,^5^ but no such resistance genes are present on p0056T/p0430T/p0543. Related plasmids are present in GenBank (Figure S1 (available as Supplementary data at *JAC* Online)), but none carry a *czc* operon. The backbone of these three plasmids is similar to those of pND6–2, pS04 90 (CP011370), and unnamed plasmid 1 of strain 2881 (CP116726), all originating from *P. aeruginosa*. Compared with cryptic plasmid pND6-2, proposed as a novel Inc type,^6^ RepB is identical while ParA and ParB differ by one and two amino acids, respectively. However, MOB-suite (https://github.com/phac-nml/mob-suite) assigned them to the rep_cluster_339. While the *czc* operon of the plasmid lineage identified in this study is identical to those carried by IncP, IncU and rep_cluster_1115, this is the first time this operon has been detected on a plasmid of rep_cluster_339.

The Mauve alignment (Figure S2) shows substantial differences in the transposon segments outside the central region with the *czcABC* genes. The *czcABC* operon in the plasmids studied here appeared to be within in a Tn*4661*-like transposon while the same or closely related *czcABC* operons have been seen in related (Tn*4652*-like) transposons originating in *P. aeruginosa*. This observation shows insertion of the *czc* operon and adjacent genes (Figure 1; Figure S2) within different, but related transposons. The nucleotide sequences of *czc* operons compared in this study (Figure 1) were identical, except for the one in CP118639, which is 98.2% identical.

**Figure 1.**
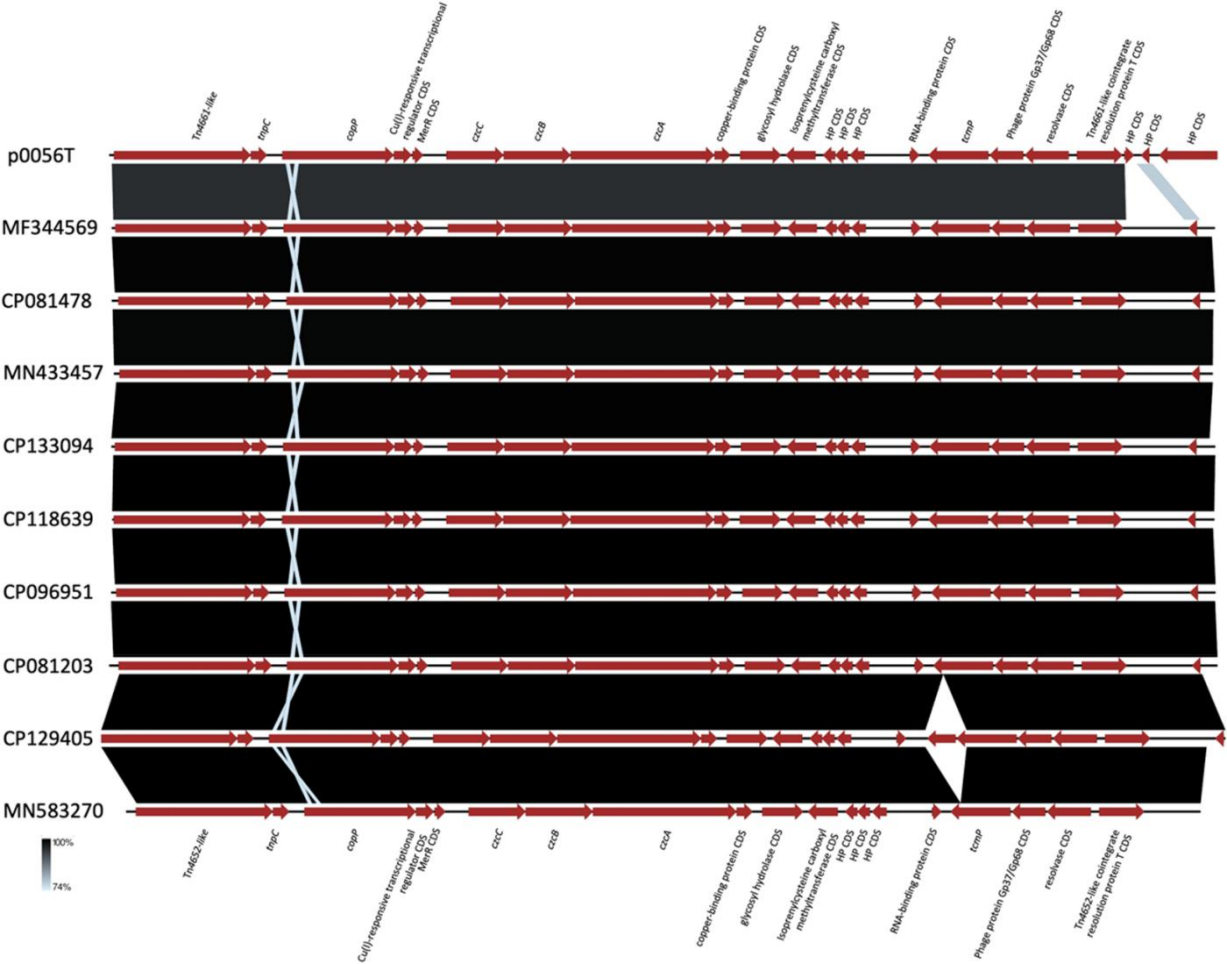
A linear nucleotide comparison generated using EasyFig (https://mjsull.github.io/Easyfig/) of Tn*4661*-like transposons that carry a *czcABC* operon of p0056T as a representative for plasmids described in this study, and plasmids with IncP, IncU and rep_cluster_1115 replicons harbouring the same operon. The plasmids in the comparison were selected on arbitrary threshold (≥97% sequence identity and ≥74% query coverage) based on BLAST search of the transposon carrying the *czc* operon in p0056T, p0430T and p0543T. The *P. aeruginosa* strain pae001 (CP133094) represents strains carrying the *czcABC* operon chromosomally.

The plasmids remained stable as they were detected in samples from different sampling dates, while the patient underwent various antibiotic courses over several months, suggesting a maintenance mechanisms or a selective pressure, such as silver sulfadiazine used in the patient. A conjugative plasmid conferring heavy-metal resistance can be especially troublesome in combination with multidrug-resistant (MDR) strains of *P. aeruginosa.* The p0056T/p0430T/p0543T plasmid carries genes involved in conjugative transfer, similar to those in pND6-2 (CP003589; Figure S1), which belongs to the same rep_cluster_339. pND6-2 was found to be capable of conjugative transfer,^6^ suggesting that p0056T, p0430T and p0543T may also be conjugative.

Infections by MDR organisms are increasingly complicating burn injuries.^7^ The use of effective topical antimicrobial agents, such as silver sulfadiazine, active against a wide spectrum of bacteria, including *A. baumannii* and *P. aeruginosa*, is a cornerstone of burn wound care.^7,8^ While burn wound swabs are taken on a regular basis and clinical microbiology laboratories perform conventional antibiotic susceptibility testing, the susceptibility of burn wound isolates to commonly used topical antimicrobial agents or heavy metals is rarely determined, mostly in research settings. In the absence of routine testing of susceptibility to commonly used topical antimicrobial agents, burn units may try rotating their use on a regular basis to reduce the risk of development of antibiotic resistance.

Alternatively, active accumulation of copper in the cytosol as a response to infection with intra- and extra-cellular bacteria may serve as selective pressure for bacteria harbouring plasmids carrying the *czc* operon.^9^ However, these hypotheses still require an experimental confirmation.

Our findings highlight the risk of a plasmid type in which the presence of the *czc* operon was not detected before. The surveillance of mobile genetic elements contributing to epidemiology and virulence is critical, yet often neglected in comparison to elements related to antibiotic resistance, especially within ESKAPE pathogens.

## Supplementary Material

dkaf058_Supplementary_Data

## Supplementary data

Figures S1 and S2 are available as Supplementary data at *JAC* Online.
