# Supplementary material for: Identification of the czc metal efflux operon on a new plasmid type in a Pseudomonas aeruginosa clinical isolate belonging to ST357 O11
Source: J Antimicrob Chemother. 2025 Mar 5;80(4):1158–60. doi: 10.1093/jac/dkaf058 (PMC11962381; doi:10.1093/jac/dkaf058)
Supplement: dkaf058_Supplementary_Data [file dkaf058_supplementary_data.docx]

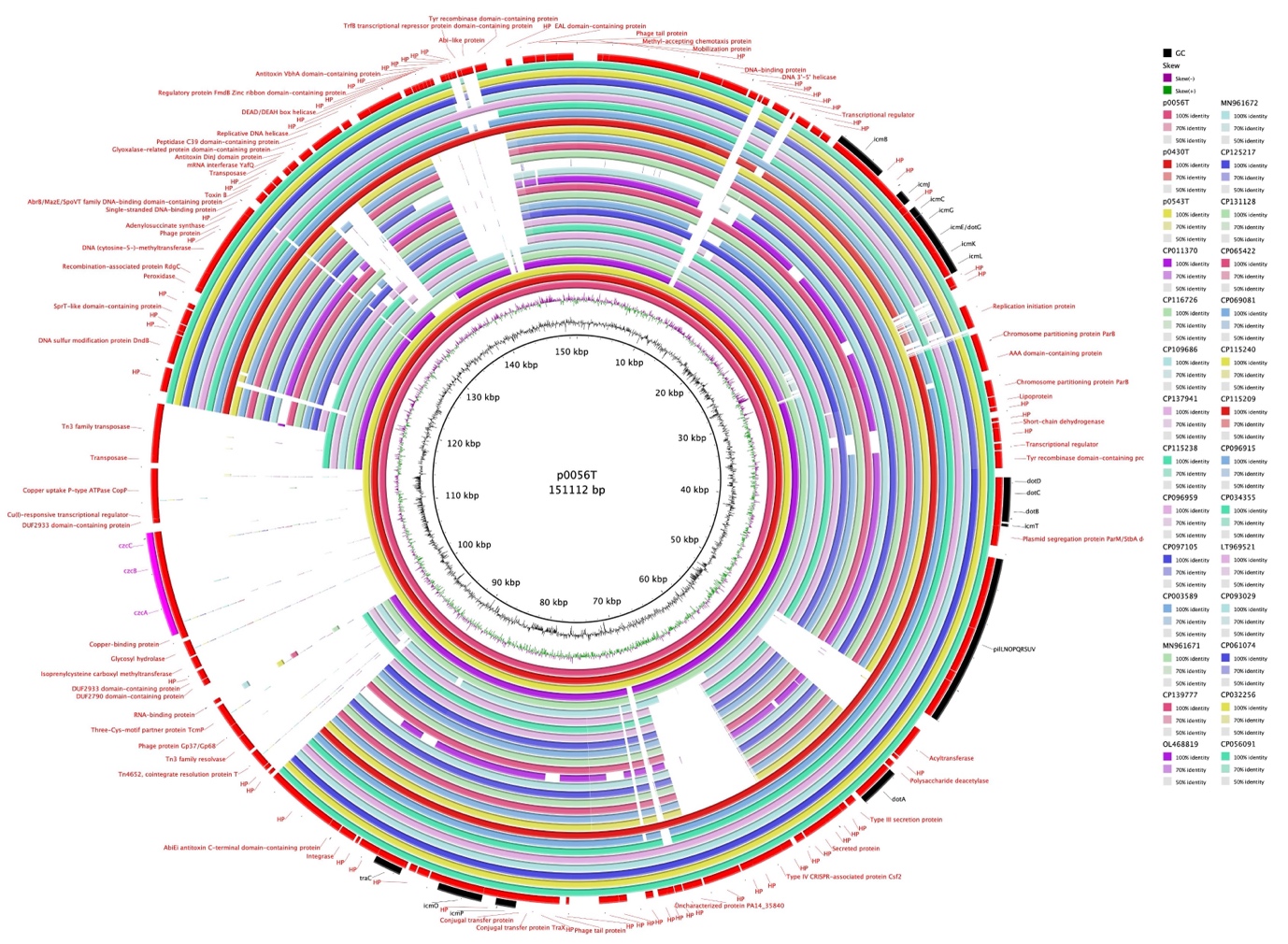


Supplementary Figure 1: Comparison of p0056T, p0430T and p0543T with rep_cluster_339 plasmids that lack the *czcABC* operon using BRIG (https://github.com/happykhan/BRIG). The genes involved in conjugative transfer, as described for pND6-2 (CP003589), are represented by a black arc, while the *czcABC* operon is represented by a fuchsia arc. The left column of the legend continues on the right, from top to the bottom.


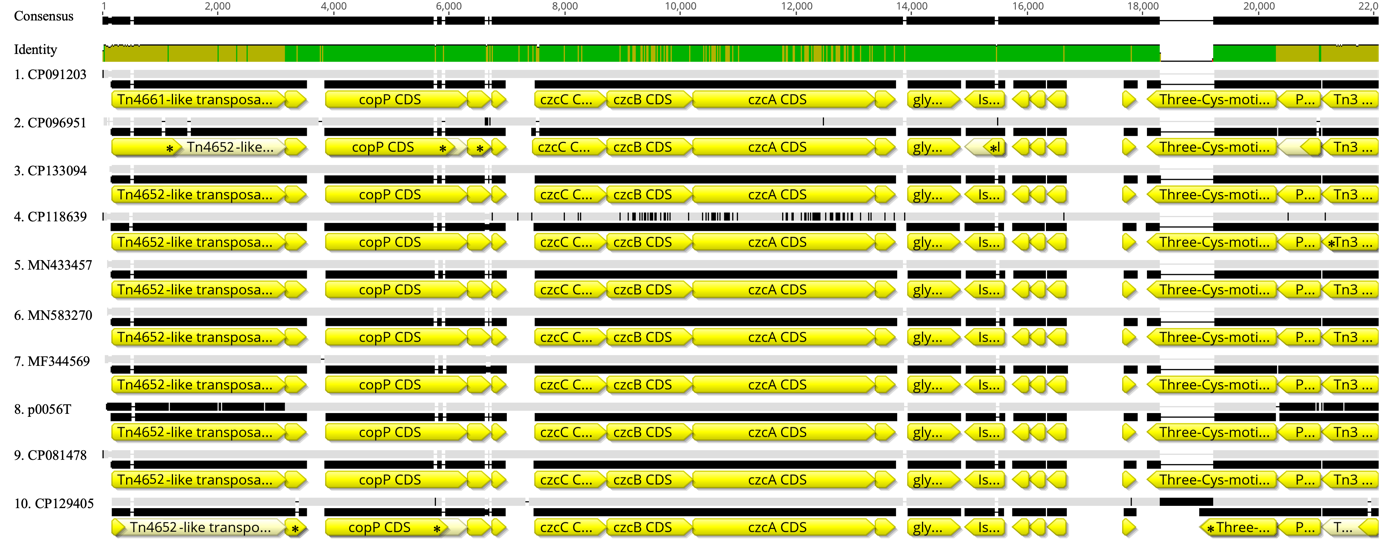


Supplementary Figure 2: A Mauve alignment showing substantial differences in the transposon segments outside the central region with a *czcABC* genes as the plasmids examined in this study carry a *czcABC* operon within Tn*4661-like* transposon, while the related plasmids carry a *czc* operon within Tn*4652-like* transposon.
